# Authors’ Response to Peer Reviews of “No Time to Waste: Real-World Repurposing of Generic Drugs as a Multifaceted Strategy Against COVID-19”

**DOI:** 10.2196/24485

**Published:** 2020-09-30

**Authors:** Moshe Rogosnitzky, Esther Berkowitz, Alejandro R Jadad

**Affiliations:** 1 Medinsight Research Institute Rehovot Israel; 2 Program in Impactful Giving Dalla Lana School of Public Health University of Toronto Toronto, ON Canada

**Keywords:** COVID-19, drug repurposing

*Author response to peer reviews for “No Time to Waste: Real-World Repurposing of Generic Drugs as a Multifaceted Strategy Against COVID-19.”*

## Response to Round 1 Reviews

### Response to Reviewers

The authors of the manuscript are grateful to the editor and reviewers for their invaluable input and feedback. We have taken the necessary steps to address the comments given and will be providing a summary of the changes made to the manuscript here. We also ensured that we revised the manuscript in line with common formatting/editorial guidelines as listed in JMIRx’s “Instructions for Authors” guide.

#### Reviewer E

The paper is a narrative review that consolidates the extensive evidence in the literature supporting the potential efficacy of six generic drugs (as discussed in the manuscript) in the management of COVID-19 patients. As such, it does not align with the “Introduction, Methods, Results, and Discussion-IMRD” structure that will be expected from an original article. Also, the current structure of the manuscript is in line with similar narrative reviews already published on JMIRx.

#### Reviewer F

##### Overall

All nonstandard abbreviations have been removed from the manuscript and use of abbreviations reduced significantly. Also, the authors have ensured that each abbreviation used is well defined at the point of first use.

We have included new papers (clinical trials) that have been generated since the manuscript was first submitted, as recommended.

##### Abstract

We have included the drug class for each drug and summarized the pathophysiological mechanism through which the drugs may help improve treatment outcomes in patients with COVID-19.

##### Background

Similar to the changes made to the abstract, we also explicitly mentioned the class to which each of the six drugs belongs as well as a summary of their underlying pathophysiological mechanism.

##### Pathophysiology of COVID-19 section

We have stated explicitly that the percentages reported are from hospitalized patients rather than individuals in the community.

We have also included relevant references in the table and changed the orientation of the table, as recommended.

As suggested, we have listed the major plasma inflammatory biomarkers that are abnormal in COVID-19, and these are the same as listed in Table 1.

##### Potential Therapies Within the Current Pharmacopeia section

We have restructured the sentence starting with “All three drugs...” to improve clarity and comprehension.

With respect to Table 2, we have added references from which the data presented for each drug have been sourced.

##### Dipyridamole section

We added a reference for the sentence starting with “Within the 200-400 mg....”

We have restructured the sentence starting with “Examples include...,” first to improve the overall clarity of the sentence, and second to provide an explanation for “widening the therapeutic window of glucocorticoid activity.”

##### Sildenafil section

We have included trial numbers/identifiers in Table 2 for relevant ongoing clinical trials relating to sildenafil.

##### Conclusions

We modified the sentence beginning with “The efforts we make now to facilitate...” to improve its overall clarity.

##### Other minor comments

We made the headings for Sildenafil and Fenofibrate / Bezafibrate bold for consistency with other similar headings.

The “V” in SARS-CoV-2 has been capitalized in all cases.

All page numbers are now sequential.

